# Supplementary material for: Spodoptera frugiperda Uses Specific Volatiles to Assess Maize Development for Optimal Offspring Survival
Source: Insects. 2025 Jun 4;16(6):592. doi: 10.3390/insects16060592 (PMC12193326; doi:10.3390/insects16060592)
Supplement: Supplementary file 1 [file insects-16-00592-s001.zip › insects-3594761-supplementary.pdf]

Supplementary Materials

Spodoptera frugiperda Uses Specific Volatiles to Assess Maize Development for Optimal Offspring Survival

Hanbing Li [1, 2], Peng Wan [1], Zhihui Zhu [2], Dong Xu [1], Shengbo Cong [1], Min Xu [1] and Haichen Yin [1*]

[1] Central China Key Laboratory of Integrated Pest Management, Ministry of Agriculture and Rural Affairs , Hubei Key Laboratory of Major Crop Diseases, Pests and Weeds Prevention and Control, Institute of Plant Protection and Soil Fertilizer, Hubei Academy of Agricultural Sciences, Wuhan 430064, China; 18331202139@163.com (H.L.); wanpenghb@126.com (P.W.); ztb799@163.com (D.X.); congshengbo@163.com (S.C.); xumin94@mails.ccnu.edu.cn (M.X.)

2 Hubei Insect Resources Utilization and Sustainable Pest Management Key Laboratory, College of Plant Science & Technology , Huazhong Agricultural University, Wuhan 430070, China; zhi-hui@mail.hzau.edu.cn

* Correspondence: 15827546601@163.com

Supplementary Materials Figure S1

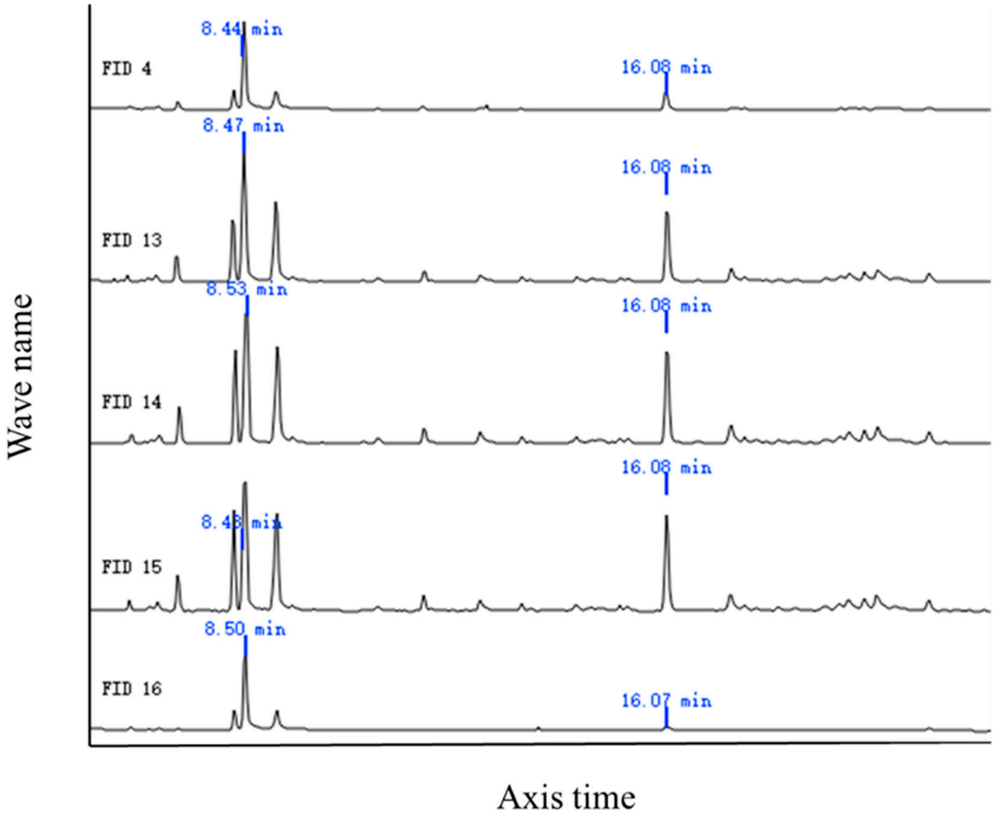

GC-EAD response of female *S. frugiperda* to corn volatiles

The blue numbers in the figure represent the retention time of active components that cause electrophysiological responses in female *S. frugiperda.*

Supplementary Table S1

The content of volatile components in maize at SS stage

Retention time (min)	Area	Area percentage (%)	Peak height	Percentage of peak (%)
14.764	24312	0.10	5122	0.04
14.952	32439	0.13	5384	0.04
15.116	4221	0.02	1326	0.01
15.276	22626	0.09	6021	0.04
15.412	26541	0.10	5754	0.04
15.887	718	0.00	273	0.00
15.941	290	0.00	184	0.00
16.102	1107688	4.34	267385	1.89
16.507	4778	0.02	1027	0.01
16.708	13547	0.05	1534	0.01
16.916	1727	0.01	423	0.00
17.112	1155	0.00	340	0.00
17.294	133420	0.52	19937	0.14
17.521	24505	0.10	6430	0.05
17.629	2547	0.01	825	0.01
17.756	7200	0.03	1438	0.01
17.899	8493	0.03	2135	0.02
18.130	3225	0.01	924	0.01
18.169	1819	0.01	376	0.00
18.413	1255	0.00	341	0.00
18.491	7943	0.03	1248	0.01
18.705	5945	0.02	1603	0.01
18.866	1564	0.01	597	0.00
19.017	15150	0.06	3156	0.02
19.109	1569	0.01	783	0.01
19.252	21838	0.09	5524	0.04
19.443	71207	0.28	9378	0.07
19.682	70746	0.28	18953	0.13
20.038	144647	0.57	11646	0.08
20.231	7174	0.03	1861	0.01
20.302	3371	0.01	611	0.00
20.843	147553	0.58	25046	0.18
21.132	12333	0.05	2354	0.02
21.329	982	0.00	382	0.00
21.384	3161	0.01	970	0.01
21.588	3720	0.01	924	0.01
21.720	1193	0.00	392	0.00
21.846	5253	0.02	1126	0.01
22.061	2392	0.01	538	0.00
22.153	3356	0.01	568	0.00

22.317	459	0.00	180	0.00
22.595	5143	0.02	1236	0.01
22.777	56649	0.22	10085	0.07
23.137	26074	0.10	2809	0.02
23.308	13646	0.05	3327	0.02
23.487	498	0.00	195	0.00
23.517	127	0.00	155	0.00
23.551	487	0.00	242	0.00
23.745	3331	0.01	625	0.00
23.838	1364	0.01	468	0.00
23.911	671	0.00	277	0.00
24.120	14780	0.06	2213	0.02
24.372	29372	0.11	4312	0.03
24.509	1332	0.01	370	0.00
24.666	29203	0.11	5581	0.04
25.071	13956	0.05	2325	0.02
25.197	128366	0.50	33777	0.24
25.293	5064	0.02	1514	0.01
25.403	4363	0.02	1468	0.01
25.527	361	0.00	214	0.00
25.720	78363	0.31	13204	0.09
25.968	2651	0.01	716	0.01
26.054	1747	0.01	620	0.00
26.159	34629	0.14	9097	0.06
26.361	10868	0.04	2230	0.02
26.541	2842	0.01	1012	0.01
26.602	2472	0.01	752	0.01
26.754	13638	0.05	2211	0.02
26.968	3601	0.01	1224	0.01
27.074	352	0.00	240	0.00
27.156	686	0.00	310	0.00
27.289	30714	0.12	7475	0.05
27.413	2586	0.01	677	0.00
27.568	12951	0.05	2301	0.02
27.868	16311	0.06	3655	0.03
28.090	6162	0.02	1560	0.01
28.150	2078	0.01	882	0.01
28.251	2714	0.01	1073	0.01
28.333	27627	0.11	8717	0.06
28.485	2919	0.01	1133	0.01
28.564	5990	0.02	2519	0.02
28.672	11522	0.05	3564	0.03
28.872	6726	0.03	2329	0.02
29.007	137220	0.54	28738	0.20

29.131	6864	0.03	2678	0.02
29.210	3856	0.02	1217	0.01
29.358	6191	0.02	1751	0.01
29.505	3188	0.01	940	0.01
29.646	29090	0.11	8198	0.06
29.786	10378	0.04	2313	0.02
29.920	3028	0.01	1232	0.01
30.017	9824	0.04	3017	0.02
30.147	1173	0.00	489	0.00
30.247	6591	0.03	2153	0.02
30.345	7578	0.03	3195	0.02
30.438	26105	0.10	4848	0.03
30.639	18278	0.07	5713	0.04
30.736	5256	0.02	2281	0.02
30.957	25264	0.10	4496	0.03
31.131	13058	0.05	2544	0.02
31.284	14189	0.06	3889	0.03
31.438	14901	0.06	3774	0.03
31.521	10596	0.04	3960	0.03
31.616	959	0.00	755	0.01
31.653	333	0.00	297	0.00
31.698	2008	0.01	1351	0.01
31.769	37469	0.15	17099	0.12
31.837	9042	0.04	4379	0.03
31.949	999	0.00	575	0.00
32.056	2610	0.01	1573	0.01
32.105	764	0.00	531	0.00
32.159	16148	0.06	9014	0.06
32.221	18844	0.07	8419	0.06
32.300	2850	0.01	1294	0.01
32.438	17710	0.07	4710	0.03
32.558	1068	0.00	757	0.01
32.606	3093	0.01	1292	0.01
32.733	5985	0.02	2317	0.02
32.790	874	0.00	543	0.00
32.912	13457	0.05	3702	0.03
33.007	7175	0.03	2611	0.02
33.187	25736	0.10	5947	0.04
33.266	2817	0.01	1348	0.01
33.357	16991	0.07	6753	0.05
33.454	36597	0.14	14320	0.10
33.657	10217	0.04	3214	0.02
33.715	20010	0.08	9201	0.07
33.822	7072	0.03	3683	0.03

33.875	842	0.00	420	0.00
33.923	877	0.00	680	0.00
33.965	955	0.00	590	0.00
34.041	1520	0.01	829	0.01
34.082	918	0.00	631	0.00
34.145	885	0.00	503	0.00
34.219	15398	0.06	7130	0.05
34.282	771	0.00	348	0.00
34.403	4163	0.02	1088	0.01
34.503	5091	0.02	2294	0.02
34.543	1139	0.00	636	0.00
34.721	21070	0.08	5236	0.04
34.766	1841	0.01	1045	0.01
34.896	4025	0.02	2228	0.02
34.963	9870	0.04	3995	0.03
35.032	13247	0.05	6317	0.04
35.209	8313	0.03	1964	0.01
35.366	1119	0.00	338	0.00
35.500	883	0.00	629	0.00
35.593	20523	0.08	6497	0.05
35.734	1110	0.00	575	0.00
35.798	4149	0.02	1472	0.01
35.912	18690	0.07	6935	0.05
36.006	8047	0.03	3942	0.03
36.053	2173	0.01	1187	0.01
36.267	44113	0.17	6943	0.05
36.518	8949	0.04	1356	0.01
36.704	1026	0.00	463	0.00
36.941	16148	0.06	5142	0.04
37.032	22284	0.09	6768	0.05
37.157	212	0.00	294	0.00
37.178	196	0.00	187	0.00
37.319	8687	0.03	2101	0.01
37.392	579	0.00	571	0.00
37.464	478	0.00	202	0.00
37.583	2692	0.01	848	0.01
37.682	13271	0.05	3999	0.03
37.768	1704	0.01	897	0.01
37.932	63721	0.25	12994	0.09
38.203	18792	0.07	4180	0.03
38.334	12624	0.05	3537	0.03
38.417	201	0.00	286	0.00
38.438	138	0.00	171	0.00
38.479	183	0.00	187	0.00

38.569	2556	0.01	596	0.00
38.653	256	0.00	156	0.00
38.694	1026	0.00	215	0.00
38.843	921	0.00	349	0.00
39.005	37209	0.15	7354	0.05
39.208	251	0.00	172	0.00
39.261	433	0.00	390	0.00
39.402	51721	0.20	7867	0.06
39.635	293	0.00	291	0.00
39.803	85116	0.33	11279	0.08
40.068	2146	0.01	507	0.00
40.317	26316	0.10	3643	0.03
40.542	278	0.00	224	0.00
40.651	2685	0.01	557	0.00
40.786	449	0.00	290	0.00
40.821	1170	0.00	323	0.00
41.028	104	0.00	119	0.00
41.057	118	0.00	155	0.00
41.097	263	0.00	195	0.00
41.279	5984	0.02	865	0.01
41.415	205	0.00	259	0.00
41.459	163	0.00	223	0.00
41.525	126	0.00	7	0.00
41.567	150	0.00	191	0.00
41.602	124	0.00	123	0.00
41.632	293	0.00	189	0.00
41.682	188	0.00	126	0.00
41.729	95	0.00	231	0.00
41.935	36357	0.14	4567	0.03
42.137	218	0.00	205	0.00
42.180	107	0.00	96	0.00
42.212	327	0.00	188	0.00
42.263	133	0.00	118	0.00
42.288	213	0.00	174	0.00
42.494	6914	0.03	796	0.01
42.649	342	0.00	194	0.00
42.690	234	0.00	215	0.00
42.718	209	0.00	192	0.00
42.898	121	0.00	193	0.00
43.103	10404	0.04	1352	0.01
43.238	244	0.00	142	0.00
43.271	135	0.00	232	0.00
43.297	282	0.00	237	0.00
43.324	178	0.00	209	0.00

43.358	179	0.00	214	0.00
43.379	375	0.00	222	0.00
43.420	164	0.00	186	0.00
43.448	335	0.00	211	0.00
43.486	158	0.00	193	0.00
43.512	742	0.00	344	0.00
43.618	734	0.00	275	0.00
43.775	1630	0.01	448	0.00
43.911	16632	0.07	1807	0.01
44.078	437	0.00	383	0.00
44.109	666	0.00	351	0.00
44.154	438	0.00	315	0.00
44.173	292	0.00	319	0.00
44.208	762	0.00	351	0.00
44.241	283	0.00	436	0.00
44.266	603	0.00	388	0.00
44.288	434	0.00	444	0.00
44.352	2085	0.01	477	0.00
44.429	1010	0.00	502	0.00
44.453	861	0.00	494	0.00
44.502	837	0.00	436	0.00
44.770	21929	0.09	1439	0.01
45.038	411	0.00	161	0.00
45.068	394	0.00	253	0.00
45.088	447	0.00	294	0.00
45.148	659	0.00	289	0.00
45.179	373	0.00	319	0.00
45.290	1592	0.01	383	0.00
45.374	2001	0.01	553	0.00
45.410	897	0.00	547	0.00
45.462	2311	0.01	570	0.00
45.902	27718	0.11	2149	0.02
45.959	9459	0.04	2263	0.02
45.989	5638	0.02	2244	0.02
46.048	4405	0.02	2307	0.02
46.078	8015	0.03	2247	0.02
46.130	9715	0.04	2065	0.01
46.228	6998	0.03	1834	0.01
46.331	20570	0.08	1774	0.01
46.597	854	0.00	385	0.00
46.655	1282	0.01	418	0.00
46.730	230	0.00	205	0.00
46.764	442	0.00	242	0.00
46.827	291	0.00	269	0.00

46.888	747	0.00	263	0.00
46.937	653	0.00	250	0.00
46.989	193	0.00	184	0.00
47.024	313	0.00	257	0.00
47.052	381	0.00	248	0.00
47.112	601	0.00	229	0.00
47.167	605	0.00	276	0.00
47.229	561	0.00	280	0.00
47.313	246	0.00	203	0.00
47.342	176	0.00	176	0.00
47.376	314	0.00	164	0.00
47.448	313	0.00	200	0.00
47.471	401	0.00	198	0.00
47.554	332	0.00	199	0.00
47.593	441	0.00	234	0.00
47.632	596	0.00	282	0.00
47.689	411	0.00	333	0.00
47.725	715	0.00	282	0.00
47.803	750	0.00	276	0.00
47.859	769	0.00	1749	0.01
47.897	183	0.00	211	0.00
47.939	979	0.00	293	0.00
48.026	956	0.00	385	0.00
48.103	775	0.00	334	0.00
48.135	1378	0.01	380	0.00
48.216	409	0.00	330	0.00
48.242	328	0.00	276	0.00
48.286	282	0.00	210	0.00
48.340	299	0.00	204	0.00
48.384	226	0.00	173	0.00
48.503	801	0.00	210	0.00
48.589	781	0.00	279	0.00
48.657	184	0.00	102	0.00
48.688	538	0.00	174	0.00
48.788	163	0.00	218	0.00
48.807	187	0.00	147	0.00
48.847	324	0.00	249	0.00
48.894	300	0.00	214	0.00

Supplementary Table S2

The content of volatile components in maize at STS stage

Retention time (min)	Area	Area percentage (%)	Peak height	Percentage of peak (%)
14.775	14577	0.04	2946	0.01
14.963	36361	0.09	6556	0.03
15.168	15428	0.04	4819	0.02
15.277	13066	0.03	3829	0.02
15.408	37537	0.09	7748	0.03
15.831	314	0.00	156	0.00
15.870	143	0.00	167	0.00
15.969	1263	0.00	394	0.00
16.106	581083	1.41	199418	0.80
16.188	322290	0.78	114664	0.46
16.311	5258	0.01	1618	0.01
16.502	7745	0.02	1453	0.01
16.683	81	0.00	164	0.00
16.782	4650	0.01	826	0.00
16.933	2540	0.01	619	0.00
17.166	153	0.00	189	0.00
17.313	57903	0.14	8550	0.03
17.536	11358	0.03	2909	0.01
17.630	3536	0.01	1051	0.00
17.832	9893	0.02	1729	0.01
17.906	14898	0.04	3642	0.01
18.126	7840	0.02	1781	0.01
18.448	362	0.00	200	0.00
18.497	403	0.00	214	0.00
18.619	3701	0.01	996	0.00
18.718	4550	0.01	1191	0.00
18.873	1392	0.00	500	0.00
19.021	16188	0.04	3406	0.01
19.115	1253	0.00	459	0.00
19.254	20534	0.05	4874	0.02
19.408	2098	0.01	632	0.00
19.526	7245	0.02	1778	0.01
19.686	61019	0.15	15040	0.06
19.907	48809	0.12	5899	0.02
20.062	7554	0.02	2283	0.01
20.349	149719	0.36	11132	0.04
20.854	1531664	3.71	363486	1.46
21.142	13613	0.03	2625	0.01
21.381	11274	0.03	1853	0.01
21.597	6593	0.02	1208	0.00

21.737	896	0.00	375	0.00
21.761	7	0.00	0	0.00
21.778	90	0.00	67	0.00
21.864	5307	0.01	1198	0.00
22.059	3390	0.01	780	0.00
22.180	4553	0.01	1076	0.00
22.321	227	0.00	166	0.00
22.453	1534	0.00	390	0.00
22.618	2162	0.01	478	0.00
22.785	63367	0.15	10417	0.04
23.157	25112	0.06	4277	0.02
23.310	8898	0.02	2295	0.01
23.533	64278	0.16	5043	0.02
23.925	2644	0.01	801	0.00
24.103	19461	0.05	2315	0.01
24.421	72200	0.18	6234	0.02
24.637	2008	0.00	387	0.00
25.209	1095609	2.66	278319	1.11
25.314	10365	0.03	1128	0.00
25.528	787	0.00	457	0.00
25.694	34923	0.08	8790	0.04
25.792	1987	0.00	264	0.00
25.957	3030	0.01	920	0.00
26.158	66516	0.16	12184	0.05
26.370	11855	0.03	2628	0.01
26.538	5294	0.01	1744	0.01
26.590	3516	0.01	534	0.00
26.763	6038	0.01	1429	0.01
26.844	1655	0.00	456	0.00
26.975	17502	0.04	4915	0.02
27.081	161	0.00	204	0.00
27.142	1491	0.00	490	0.00
27.291	29156	0.07	6747	0.03
27.408	892	0.00	265	0.00
27.572	7651	0.02	1800	0.01
27.722	2053	0.00	884	0.00
27.804	8265	0.02	1609	0.01
27.971	3360	0.01	1274	0.01
28.080	13364	0.03	2398	0.01
28.336	731883	1.77	232047	0.93
28.503	9671	0.02	2436	0.01
28.663	2064	0.01	689	0.00
28.736	3552	0.01	1019	0.00
28.876	10230	0.02	3690	0.01

28.948	590	0.00	413	0.00
29.008	13350	0.03	5043	0.02
29.139	2508	0.01	883	0.00
29.197	2889	0.01	1017	0.00
29.348	4581	0.01	1334	0.01
29.426	1252	0.00	569	0.00
29.470	1003	0.00	180	0.00
29.644	19721	0.05	5587	0.02
29.783	9330	0.02	2236	0.01
29.922	4209	0.01	1514	0.01
30.013	1187	0.00	568	0.00
30.093	2563	0.01	1008	0.00
30.168	521	0.00	253	0.00
30.251	1251	0.00	631	0.00
30.342	3654	0.01	1170	0.00
30.457	6709	0.02	1818	0.01
30.644	663813	1.61	219880	0.88
30.867	7097	0.02	2640	0.01
30.968	11797	0.03	2669	0.01
31.170	12809	0.03	3768	0.02
31.279	27014	0.07	8160	0.03
31.389	1790	0.00	973	0.00
31.460	1416	0.00	594	0.00
31.533	4600	0.01	1471	0.01
31.693	728	0.00	416	0.00
31.771	4192	0.01	2045	0.01
31.842	7848	0.02	2125	0.01
31.945	367	0.00	261	0.00
32.068	2618	0.01	882	0.00
32.226	642407	1.56	278355	1.11
32.373	570	0.00	322	0.00
32.440	4597	0.01	1346	0.01
32.555	1265	0.00	554	0.00
32.655	2840	0.01	1068	0.00
32.728	2556	0.01	1401	0.01
32.798	1109	0.00	485	0.00
32.876	1784	0.00	582	0.00
33.060	7622	0.02	1555	0.01
33.186	14730	0.04	3640	0.01
33.350	595742	1.44	285691	1.14
33.458	1289	0.00	624	0.00
33.508	1691	0.00	660	0.00
33.653	2751	0.01	628	0.00
33.718	4145	0.01	2040	0.01

33.830	1968	0.00	1019	0.00
33.979	1738	0.00	1002	0.00
34.103	3694	0.01	691	0.00
34.219	646779	1.57	325376	1.30
34.399	4628	0.01	1547	0.01
34.544	4011	0.01	1780	0.01
34.623	859	0.00	416	0.00
34.705	814	0.00	359	0.00
34.784	4840	0.01	1178	0.00
35.032	771993	1.87	319686	1.28
35.156	163	0.00	183	0.00
35.199	706	0.00	306	0.00
35.377	4157	0.01	1462	0.01
35.459	475	0.00	275	0.00
35.509	336	0.00	232	0.00
35.596	6122	0.01	1141	0.00
35.803	757	0.00	324	0.00
35.907	873149	2.12	317535	1.27
36.008	23241	0.06	8151	0.03
36.167	229	0.00	151	0.00
36.314	8607	0.02	1550	0.01
36.494	388	0.00	219	0.00
36.519	95	0.00	58	0.00
36.593	1658	0.00	478	0.00
36.693	699	0.00	281	0.00
36.732	250	0.00	182	0.00
36.932	938955	2.28	265068	1.06
37.138	278	0.00	132	0.00
37.184	248	0.00	241	0.00
37.218	294	0.00	227	0.00
37.307	950	0.00	288	0.00
37.367	82	0.00	142	0.00
37.471	2313	0.01	786	0.00
37.568	4457	0.01	1203	0.00
37.672	210	0.00	286	0.00
37.758	83	0.00	190	0.00
37.771	168	0.00	104	0.00
37.825	201	0.00	141	0.00
37.994	1946	0.00	328	0.00
38.017	76	0.00	77	0.00
38.198	881913	2.14	190024	0.76
38.444	170	0.00	149	0.00
38.490	375	0.00	213	0.00
38.536	214	0.00	184	0.00

38.566	266	0.00	161	0.00
38.625	247	0.00	200	0.00
38.720	426	0.00	216	0.00
38.757	140	0.00	227	0.00
38.792	46	0.00	168	0.00
38.874	308	0.00	178	0.00
38.987	5945	0.01	1124	0.00
39.101	147	0.00	208	0.00
39.132	119	0.00	191	0.00
39.232	271	0.00	279	0.00
39.248	592	0.00	236	0.00
39.346	1238	0.00	305	0.00
39.454	298	0.00	157	0.00
39.506	403	0.00	232	0.00
39.577	212	0.00	182	0.00
39.603	167	0.00	148	0.00
39.818	729119	1.77	122151	0.49
40.024	94	0.00	167	0.00
40.099	471	0.00	186	0.00
40.153	115	0.00	164	0.00
40.182	213	0.00	177	0.00
40.222	76	0.00	138	0.00
40.271	405	0.00	236	0.00
40.305	658	0.00	209	0.00
40.407	301	0.00	264	0.00
40.468	175	0.00	156	0.00
40.527	293	0.00	202	0.00
40.542	81	0.00	187	0.00
40.614	173	0.00	145	0.00
40.654	201	0.00	271	0.00
40.688	216	0.00	145	0.00
40.761	493	0.00	199	0.00
40.805	216	0.00	300	0.00
40.890	129	0.00	195	0.00
41.027	1114	0.00	307	0.00
41.169	743	0.00	216	0.00
41.260	184	0.00	165	0.00
41.282	207	0.00	189	0.00
41.321	68	0.00	99	0.00
41.347	143	0.00	136	0.00
41.400	229	0.00	197	0.00
41.446	218	0.00	196	0.00
41.493	420	0.00	238	0.00
41.552	655	0.00	215	0.00

41.631	142	0.00	165	0.00
41.663	128	0.00	163	0.00
41.688	245	0.00	223	0.00
41.944	542671	1.32	65700	0.26
42.202	274	0.00	184	0.00
42.233	106	0.00	167	0.00
42.275	237	0.00	146	0.00
42.316	228	0.00	210	0.00
42.342	276	0.00	259	0.00
42.397	774	0.00	300	0.00
42.434	230	0.00	181	0.00
42.472	184	0.00	127	0.00
42.512	184	0.00	157	0.00
42.555	109	0.00	117	0.00
42.601	410	0.00	193	0.00
42.712	643	0.00	411	0.00
42.767	436	0.00	218	0.00
42.801	186	0.00	181	0.00
42.861	194	0.00	193	0.00
42.886	183	0.00	170	0.00
42.996	777	0.00	224	0.00
43.050	476	0.00	260	0.00
43.114	558	0.00	230	0.00
43.273	601	0.00	268	0.00
43.299	309	0.00	190	0.00
43.364	581	0.00	226	0.00
43.403	383	0.00	284	0.00
43.448	558	0.00	290	0.00
43.505	370	0.00	257	0.00
43.532	549	0.00	265	0.00
43.585	280	0.00	316	0.00
43.613	339	0.00	279	0.00
43.700	699	0.00	332	0.00
43.828	1777	0.00	403	0.00
43.873	1120	0.00	467	0.00
43.898	1051	0.00	479	0.00
43.953	911	0.00	399	0.00
43.983	442	0.00	358	0.00
44.121	3239	0.01	520	0.00
44.195	1357	0.00	510	0.00
44.271	2362	0.01	546	0.00
44.307	816	0.00	577	0.00
44.332	730	0.00	486	0.00
44.378	1554	0.00	520	0.00

44.441	1209	0.00	542	0.00
44.503	1480	0.00	534	0.00
44.773	378441	0.92	33300	0.13
45.050	579	0.00	373	0.00
45.081	522	0.00	371	0.00
45.114	357	0.00	275	0.00
45.162	686	0.00	237	0.00
45.231	172	0.00	191	0.00
45.280	435	0.00	212	0.00
45.353	156	0.00	161	0.00
45.498	902	0.00	218	0.00
45.595	252	0.00	226	0.00
45.618	343	0.00	212	0.00
45.656	181	0.00	190	0.00
45.716	382	0.00	201	0.00
45.769	442	0.00	241	0.00
45.839	486	0.00	230	0.00
45.867	301	0.00	218	0.00
45.938	368	0.00	215	0.00
45.974	467	0.00	286	0.00
46.012	464	0.00	205	0.00
46.077	253	0.00	228	0.00
46.121	248	0.00	198	0.00
46.157	238	0.00	239	0.00
46.176	518	0.00	211	0.00
46.242	209	0.00	248	0.00
46.278	669	0.00	275	0.00
46.368	946	0.00	309	0.00
46.406	650	0.00	295	0.00
46.489	285	0.00	264	0.00
46.518	439	0.00	324	0.00
46.556	485	0.00	311	0.00
46.578	399	0.00	200	0.00
46.629	632	0.00	304	0.00
46.727	719	0.00	312	0.00
46.753	310	0.00	244	0.00
46.786	234	0.00	167	0.00
46.852	185	0.00	166	0.00
46.879	148	0.00	145	0.00
46.924	438	0.00	212	0.00
46.971	263	0.00	188	0.00
47.007	83	0.00	147	0.00
47.043	361	0.00	238	0.00
47.077	523	0.00	255	0.00

47.173	517	0.00	272	0.00
47.241	611	0.00	278	0.00
47.264	181	0.00	236	0.00
47.298	566	0.00	234	0.00
47.335	345	0.00	231	0.00
47.412	511	0.00	245	0.00
47.452	370	0.00	215	0.00
47.481	289	0.00	300	0.00
47.534	517	0.00	232	0.00
47.639	676	0.00	224	0.00
47.681	350	0.00	254	0.00
47.718	162	0.00	187	0.00
47.742	196	0.00	167	0.00

Supplementary Table S3

The content of volatile components in maize at FS stage

Retention time (min)	Area	Area percentage (%)	Peak height	Percentage of peak (%)
14.835	926	0.00	308	0.00
14.972	5899	0.02	1818	0.01
15.071	18093	0.07	4440	0.02
15.283	4477	0.02	1165	0.01
15.417	19467	0.08	3069	0.02
15.788	358	0.00	119	0.00
15.939	1722	0.01	428	0.00
16.107	645405	2.50	173543	0.93
16.323	6247	0.02	1073	0.01
16.756	4877	0.02	1124	0.01
16.923	5820	0.02	1227	0.01
17.172	274	0.00	204	0.00
17.335	36191	0.14	4907	0.03
17.536	12952	0.05	1264	0.01
17.918	11837	0.05	1667	0.01
18.102	256	0.00	175	0.00
18.133	240	0.00	196	0.00
18.239	9123	0.04	1889	0.01
18.436	87	0.00	156	0.00
18.467	190	0.00	251	0.00
18.490	108	0.00	127	0.00
18.644	10795	0.04	1833	0.01
19.035	23101	0.09	2481	0.01
19.237	6067	0.02	1374	0.01
19.403	7539	0.03	808	0.00
19.681	3909	0.02	977	0.01

19.862	23726	0.09	3538	0.02
20.045	178	0.00	147	0.00
20.112	95	0.00	176	0.00
20.377	72445	0.28	5811	0.03
20.833	64366	0.25	6151	0.03
21.110	930	0.00	179	0.00
21.300	1037	0.00	268	0.00
21.352	763	0.00	109	0.00
21.514	284	0.00	152	0.00
21.567	1132	0.00	196	0.00
21.719	284	0.00	172	0.00
21.779	568	0.00	240	0.00
21.884	693	0.00	199	0.00
21.939	130	0.00	109	0.00
21.990	77	0.00	139	0.00
22.128	443	0.00	238	0.00
22.161	740	0.00	223	0.00
22.248	188	0.00	219	0.00
22.338	3415	0.01	837	0.00
22.453	157	0.00	191	0.00
22.635	7790	0.03	1896	0.01
22.797	6495	0.03	1121	0.01
23.011	1445	0.01	412	0.00
23.166	8320	0.03	1809	0.01
23.307	3145	0.01	703	0.00
23.538	38884	0.15	4066	0.02
23.810	5056	0.02	1297	0.01
23.910	986	0.00	335	0.00
24.082	917	0.00	296	0.00
24.152	1577	0.01	400	0.00
24.458	64883	0.25	3988	0.02
24.916	365	0.00	180	0.00
25.128	3539	0.01	1264	0.01
25.195	5826	0.02	1756	0.01
25.332	4037	0.02	985	0.01
25.533	5368	0.02	1629	0.01
25.629	2414	0.01	596	0.00
25.778	1084	0.00	285	0.00
25.964	1434	0.01	423	0.00
26.192	24343	0.09	2428	0.01
26.404	282	0.00	184	0.00
26.447	126	0.00	132	0.00
26.517	1669	0.01	488	0.00
26.620	1609	0.01	381	0.00

26.834	11187	0.04	1071	0.01
27.174	1514	0.01	380	0.00
27.296	10761	0.04	2266	0.01
27.428	138	0.00	198	0.00
27.576	2111	0.01	573	0.00
27.622	324	0.00	168	0.00
27.813	6650	0.03	1196	0.01
27.974	1697	0.01	616	0.00
28.078	6524	0.03	1136	0.01
28.241	189	0.00	200	0.00
28.333	6314	0.02	1745	0.01
28.507	8544	0.03	2127	0.01
28.666	1468	0.01	637	0.00
28.739	3112	0.01	835	0.00
28.954	3821	0.01	685	0.00
29.001	543	0.00	204	0.00
29.185	11407	0.04	1777	0.01
29.353	12785	0.05	1595	0.01
29.642	2015	0.01	559	0.00
29.778	11663	0.05	1317	0.01
30.005	220	0.00	87	0.00
30.312	4362	0.02	539	0.00
30.464	1248	0.00	458	0.00
30.514	430	0.00	154	0.00
30.640	855	0.00	468	0.00
30.716	4739	0.02	1436	0.01
30.865	49033	0.19	15348	0.08
30.969	5258	0.02	1324	0.01
31.173	14252	0.06	3942	0.02
31.288	7565	0.03	1845	0.01
31.493	608	0.00	232	0.00
31.587	4883	0.02	944	0.01
31.691	401	0.00	197	0.00
31.831	4109	0.02	721	0.00
31.889	486	0.00	294	0.00
31.934	160	0.00	146	0.00
32.153	2346	0.01	473	0.00
32.229	8864	0.03	2415	0.01
32.393	434	0.00	180	0.00
32.458	1365	0.01	494	0.00
32.562	613	0.00	334	0.00
32.631	1149	0.00	380	0.00
32.733	1240	0.00	553	0.00
32.817	618	0.00	294	0.00

32.903	898	0.00	388	0.00
33.083	3424	0.01	508	0.00
33.128	183	0.00	150	0.00
33.193	1012	0.00	338	0.00
33.272	181	0.00	156	0.00
33.350	1112	0.00	488	0.00
33.452	606	0.00	373	0.00
33.518	1003	0.00	372	0.00
33.663	464	0.00	406	0.00
33.723	1136	0.00	577	0.00
34.215	10815	0.04	633	0.00
34.288	401	0.00	243	0.00
34.328	27	0.00	106	0.00
34.426	781	0.00	241	0.00
34.542	1465	0.01	468	0.00
34.635	793	0.00	277	0.00
34.785	713	0.00	228	0.00
34.857	525	0.00	217	0.00
34.882	88	0.00	175	0.00
35.021	3196	0.01	653	0.00
35.101	121	0.00	149	0.00
35.140	283	0.00	205	0.00
35.192	419	0.00	208	0.00
35.268	422	0.00	213	0.00
35.367	1309	0.01	330	0.00
35.457	168	0.00	218	0.00
35.512	318	0.00	197	0.00
35.594	860	0.00	379	0.00
35.646	141	0.00	170	0.00
35.670	562	0.00	295	0.00
35.748	380	0.00	244	0.00
35.779	0	0.00	0	0.00
35.841	73	0.00	131	0.00
35.902	1425	0.01	502	0.00
36.015	13030	0.05	3650	0.02
36.263	679	0.00	256	0.00
36.301	350	0.00	177	0.00
36.373	365	0.00	196	0.00
36.459	441	0.00	218	0.00
36.516	285	0.00	155	0.00
36.548	252	0.00	218	0.00
36.601	112	0.00	182	0.00
36.661	511	0.00	226	0.00
36.741	477	0.00	237	0.00

36.822	726	0.00	296	0.00
36.923	1710	0.01	488	0.00
37.018	1984	0.01	454	0.00
37.143	409	0.00	211	0.00
37.173	91	0.00	153	0.00
37.283	380	0.00	238	0.00
37.325	291	0.00	188	0.00
37.373	211	0.00	145	0.00
37.563	68002	0.26	15620	0.08
37.860	258	0.00	184	0.00
38.013	3984	0.02	584	0.00
38.081	283	0.00	155	0.00
38.185	1347	0.01	320	0.00
38.323	3241	0.01	712	0.00
38.487	136	0.00	115	0.00
38.521	176	0.00	159	0.00
38.571	66	0.00	182	0.00
38.617	122	0.00	136	0.00
38.695	260	0.00	188	0.00
38.746	187	0.00	175	0.00
38.769	269	0.00	150	0.00
38.821	135	0.00	169	0.00
38.878	261	0.00	183	0.00
38.909	201	0.00	219	0.00
38.989	2549	0.01	526	0.00
39.088	296	0.00	196	0.00
39.152	101	0.00	156	0.00
39.180	313	0.00	220	0.00
39.236	340	0.00	222	0.00
39.379	1059	0.00	365	0.00
39.415	598	0.00	187	0.00
39.513	110	0.00	195	0.00
39.573	125	0.00	160	0.00
39.594	131	0.00	206	0.00
39.632	169	0.00	145	0.00
39.773	1609	0.01	371	0.00
39.836	270	0.00	118	0.00
39.884	290	0.00	200	0.00
39.931	245	0.00	292	0.00
39.970	643	0.00	194	0.00
40.081	310	0.00	183	0.00
40.135	212	0.00	207	0.00
40.162	198	0.00	149	0.00
40.229	398	0.00	196	0.00

40.294	193	0.00	195	0.00
40.350	105	0.00	201	0.00
40.437	381	0.00	180	0.00
40.504	263	0.00	244	0.00
40.562	324	0.00	217	0.00
40.625	401	0.00	187	0.00
40.662	361	0.00	173	0.00
40.717	167	0.00	138	0.00
40.757	146	0.00	186	0.00
40.803	266	0.00	223	0.00
40.862	406	0.00	225	0.00
40.901	303	0.00	160	0.00
40.946	311	0.00	214	0.00
41.059	1019	0.00	292	0.00
41.129	241	0.00	185	0.00
41.165	175	0.00	143	0.00
41.189	78	0.00	70	0.00
41.228	214	0.00	188	0.00
41.254	166	0.00	176	0.00
41.328	370	0.00	208	0.00
41.362	102	0.00	252	0.00
41.432	213	0.00	232	0.00
41.473	161	0.00	191	0.00
41.503	158	0.00	169	0.00
41.537	286	0.00	153	0.00
41.573	290	0.00	191	0.00
41.618	177	0.00	181	0.00
41.648	118	0.00	162	0.00
41.698	228	0.00	150	0.00
41.739	196	0.00	169	0.00
41.807	367	0.00	167	0.00
41.855	168	0.00	157	0.00
41.897	261	0.00	179	0.00
41.934	462	0.00	215	0.00
41.988	96	0.00	118	0.00
42.055	391	0.00	199	0.00
42.124	277	0.00	172	0.00
42.186	402	0.00	176	0.00
42.270	424	0.00	193	0.00
42.360	295	0.00	215	0.00
42.431	475	0.00	162	0.00
42.477	220	0.00	201	0.00
42.523	135	0.00	156	0.00
42.548	137	0.00	188	0.00

42.612	187	0.00	158	0.00
42.673	314	0.00	242	0.00
42.714	195	0.00	179	0.00
42.745	346	0.00	243	0.00
42.811	367	0.00	264	0.00
42.848	153	0.00	166	0.00
42.877	201	0.00	167	0.00
42.923	193	0.00	214	0.00
42.998	424	0.00	169	0.00
43.028	186	0.00	162	0.00
43.069	430	0.00	237	0.00
43.123	273	0.00	243	0.00
43.157	351	0.00	236	0.00
43.199	355	0.00	215	0.00
43.248	261	0.00	195	0.00
43.282	762	0.00	194	0.00
43.391	252	0.00	177	0.00
43.455	514	0.00	203	0.00
43.503	351	0.00	228	0.00
43.537	207	0.00	245	0.00
43.603	560	0.00	356	0.00
43.668	575	0.00	255	0.00
43.709	443	0.00	256	0.00
43.853	1086	0.00	288	0.00
43.892	454	0.00	307	0.00
43.936	443	0.00	414	0.00
43.999	604	0.00	266	0.00
44.021	286	0.00	288	0.00
44.051	234	0.00	230	0.00
44.078	254	0.00	264	0.00
44.168	1207	0.00	309	0.00
44.254	1418	0.01	404	0.00
44.342	2084	0.01	413	0.00
44.397	1382	0.01	231	0.00
44.489	830	0.00	483	0.00
44.689	6626	0.03	761	0.00
44.757	2786	0.01	835	0.00
44.792	1189	0.00	906	0.00
44.805	1382	0.01	941	0.01
44.858	2198	0.01	920	0.00
44.908	3062	0.01	944	0.01
44.943	2423	0.01	890	0.00
45.047	4603	0.02	1034	0.01
45.193	7636	0.03	1126	0.01

45.298	6646	0.03	1135	0.01
45.342	2493	0.01	1140	0.01
45.373	3415	0.01	1112	0.01
45.530	11621	0.05	1202	0.01
45.595	3025	0.01	1156	0.01
45.675	5537	0.02	1247	0.01
45.727	2328	0.01	1196	0.01
45.759	505	0.00	1206	0.01
45.767	4381	0.02	1136	0.01
45.851	2705	0.01	1052	0.01
45.883	4275	0.02	1015	0.01
45.968	1818	0.01	852	0.00
46.007	952	0.00	888	0.00
46.058	2317	0.01	820	0.00
46.092	2269	0.01	737	0.00
46.150	824	0.00	559	0.00
46.195	1757	0.01	532	0.00
46.253	738	0.00	428	0.00
46.291	1166	0.00	455	0.00
46.467	254	0.00	230	0.00
46.511	719	0.00	221	0.00
46.605	222	0.00	131	0.00
46.647	307	0.00	260	0.00
46.678	374	0.00	190	0.00
46.736	187	0.00	154	0.00
46.790	645	0.00	166	0.00
46.878	323	0.00	229	0.00
46.900	432	0.00	212	0.00
46.957	248	0.00	215	0.00
46.993	266	0.00	233	0.00
47.039	192	0.00	159	0.00
47.083	342	0.00	206	0.00
47.112	267	0.00	188	0.00
47.154	233	0.00	143	0.00
47.196	97	0.00	177	0.00
47.211	131	0.00	161	0.00
47.253	442	0.00	214	0.00
47.318	107	0.00	139	0.00
47.338	108	0.00	120	0.00
47.367	195	0.00	165	0.00
47.407	227	0.00	179	0.00
47.430	211	0.00	200	0.00
47.478	361	0.00	246	0.00
47.509	131	0.00	232	0.00

47.537	297	0.00	228	0.00
47.596	513	0.00	233	0.00
47.623	326	0.00	245	0.00
47.652	382	0.00	235	0.00
47.698	386	0.00	102	0.00
47.716	391	0.00	273	0.00
47.781	575	0.00	299	0.00
47.828	755	0.00	303	0.00
47.888	210	0.00	229	0.00
47.916	469	0.00	255	0.00
47.986	977	0.00	322	0.00
48.063	369	0.00	255	0.00
48.095	218	0.00	198	0.00
48.143	737	0.00	273	0.00
48.197	524	0.00	172	0.00
48.262	238	0.00	206	0.00
48.292	636	0.00	217	0.00
48.373	177	0.00	183	0.00
48.410	449	0.00	216	0.00
48.472	199	0.00	209	0.00
48.502	942	0.00	259	0.00
48.577	287	0.00	273	0.00
48.593	751	0.00	238	0.00
48.657	235	0.00	257	0.00
48.680	380	0.00	239	0.00
48.729	227	0.00	242	0.00
48.757	173	0.00	172	0.00
48.782	305	0.00	164	0.00
48.838	362	0.00	203	0.00
48.901	339	0.00	247	0.00
48.929	274	0.00	202	0.00
48.975	1	0.00	0	0.00

Supplementary Table S4

The content of volatile components in maize at MS stage

Retention time (min)	Area	Area percentage (%)	Peak height	Percentage of peak (%)
14.786	3577	0.03	610	0.01
15.000	3598	0.03	799	0.01
15.148	11912	0.10	2436	0.03
15.292	2398	0.02	686	0.01
15.423	9293	0.07	1717	0.02
15.777	3431	0.03	609	0.01
15.919	108	0.00	540	0.01

16.017	908	0.01	291	0.00
16.126	195408	1.57	42926	0.50
16.304	2170	0.02	161	0.00
16.509	169	0.00	153	0.00
16.532	325	0.00	129	0.00
16.686	970	0.01	290	0.00
16.750	1351	0.01	626	0.01
16.844	358	0.00	140	0.00
16.946	1688	0.01	318	0.00
17.050	280	0.00	165	0.00
17.098	319	0.00	138	0.00
17.162	109	0.00	151	0.00
17.351	25683	0.21	3562	0.04
17.534	1087	0.01	387	0.00
17.598	272	0.00	208	0.00
17.623	1412	0.01	199	0.00
17.799	175	0.00	160	0.00
17.832	205	0.00	173	0.00
17.925	5750	0.05	1045	0.01
18.168	605	0.00	248	0.00
18.195	116	0.00	163	0.00
18.433	221122	1.78	17472	0.20
19.028	18723	0.15	2002	0.02
19.288	3555	0.03	748	0.01
19.422	2000	0.02	529	0.01
19.525	2183	0.02	463	0.01
19.700	7642	0.06	1879	0.02
19.872	20806	0.17	3304	0.04
20.072	1055	0.01	384	0.00
20.383	75437	0.61	5744	0.07
20.857	96342	0.77	14083	0.16
21.145	1625	0.01	228	0.00
21.337	513	0.00	227	0.00
21.389	117	0.00	119	0.00
21.410	361	0.00	162	0.00
21.541	160	0.00	190	0.00
21.616	1257	0.01	296	0.00
21.739	428	0.00	206	0.00
21.811	385	0.00	191	0.00
21.882	1084	0.01	291	0.00
21.968	507	0.00	188	0.00
22.093	249	0.00	182	0.00
22.148	282	0.00	252	0.00
22.248	508	0.00	283	0.00

22.275	324	0.00	142	0.00
22.761	32626	0.26	4369	0.05
23.003	2289	0.02	582	0.01
23.220	3881	0.03	636	0.01
23.286	120	0.00	148	0.00
23.326	1705	0.01	414	0.00
23.578	24327	0.20	2108	0.02
23.813	1942	0.02	471	0.01
23.924	353	0.00	141	0.00
24.124	8398	0.07	1212	0.01
24.500	21714	0.17	2205	0.03
24.643	1398	0.01	225	0.00
24.895	586	0.00	293	0.00
24.952	215	0.00	124	0.00
25.038	3112	0.03	1109	0.01
25.222	36809	0.30	9129	0.11
25.433	1485	0.01	388	0.00
25.557	4195	0.03	1262	0.01
25.722	27868	0.22	4247	0.05
25.967	2859	0.02	781	0.01
26.186	22330	0.18	2899	0.03
26.381	1597	0.01	442	0.01
26.468	143	0.00	149	0.00
26.547	2900	0.02	605	0.01
26.638	631	0.01	196	0.00
26.778	458	0.00	244	0.00
26.858	6762	0.05	811	0.01
27.187	811	0.01	262	0.00
27.322	10033	0.08	1331	0.02
27.489	279	0.00	1035	0.01
27.599	2275	0.02	696	0.01
27.667	107	0.00	153	0.00
27.701	202	0.00	145	0.00
27.736	158	0.00	165	0.00
27.829	6373	0.05	1224	0.01
27.988	591	0.00	314	0.00
28.083	7870	0.06	1191	0.01
28.349	19941	0.16	5482	0.06
28.528	47111	0.38	12961	0.15
28.609	2915	0.02	737	0.01
28.754	3015	0.02	861	0.01
29.014	21640	0.17	3394	0.04
29.143	1441	0.01	522	0.01
29.203	1220	0.01	344	0.00

29.355	15238	0.12	1683	0.02
29.665	4078	0.03	1206	0.01
29.802	19830	0.16	2583	0.03
30.027	753	0.01	299	0.00
30.211	1490	0.01	410	0.00
30.260	593	0.00	277	0.00
30.322	883	0.01	326	0.00
30.478	910	0.01	436	0.01
30.546	728	0.01	388	0.00
30.655	7979	0.06	3066	0.04
30.735	2874	0.02	1112	0.01
30.878	10148	0.08	3299	0.04
30.985	6266	0.05	1453	0.02
31.190	21606	0.17	6065	0.07
31.299	8603	0.07	2574	0.03
31.555	11569	0.09	1805	0.02
31.708	1049	0.01	412	0.00
31.777	3073	0.02	1639	0.02
31.846	8448	0.07	2747	0.03
31.959	419	0.00	252	0.00
32.097	2153	0.02	658	0.01
32.166	1538	0.01	915	0.01
32.230	15600	0.13	6030	0.07
32.458	4183	0.03	875	0.01
32.559	725	0.01	387	0.00
32.636	1418	0.01	421	0.00
32.821	2868	0.02	576	0.01
32.892	1146	0.01	504	0.01
32.936	157	0.00	121	0.00
33.031	1509	0.01	618	0.01
33.118	536	0.00	216	0.00
33.202	2617	0.02	656	0.01
33.277	314	0.00	273	0.00
33.355	9528	0.08	4472	0.05
33.455	2628	0.02	1280	0.01
33.522	1189	0.01	587	0.01
33.668	1776	0.01	382	0.00
33.722	1625	0.01	878	0.01
33.767	169	0.00	236	0.00
33.874	1369	0.01	352	0.00
33.938	1562	0.01	717	0.01
34.048	934	0.01	333	0.00
34.223	12277	0.10	4794	0.06
34.299	654	0.01	307	0.00

34.409	1657	0.01	635	0.01
34.548	636	0.01	308	0.00
34.667	1030	0.01	331	0.00
34.728	915	0.01	478	0.01
34.776	645	0.01	382	0.00
34.830	612	0.00	288	0.00
34.902	389	0.00	303	0.00
35.042	19350	0.16	5135	0.06
35.203	1083	0.01	325	0.00
35.323	265	0.00	225	0.00
35.383	177	0.00	275	0.00
35.402	229	0.00	173	0.00
35.461	408	0.00	274	0.00
35.487	149	0.00	117	0.00
35.518	179	0.00	222	0.00
35.547	259	0.00	204	0.00
35.597	1396	0.01	523	0.01
35.699	152	0.00	150	0.00
35.723	192	0.00	239	0.00
35.760	280	0.00	259	0.00
35.917	13857	0.11	4578	0.05
36.026	16927	0.14	4869	0.06
36.275	2065	0.02	538	0.01
36.335	1248	0.01	252	0.00
36.468	509	0.00	269	0.00
36.548	1277	0.01	312	0.00
36.675	554	0.00	267	0.00
36.737	651	0.01	278	0.00
36.787	734	0.01	178	0.00
36.943	13813	0.11	3881	0.05
37.027	704	0.01	291	0.00
37.155	215	0.00	325	0.00
37.201	153	0.00	163	0.00
37.228	91	0.00	89	0.00
37.286	976	0.01	279	0.00
37.349	479	0.00	166	0.00
37.443	250	0.00	207	0.00
37.478	255	0.00	226	0.00
37.586	3640	0.03	1010	0.01
37.666	11	0.00	87	0.00
37.722	119	0.00	145	0.00
37.757	286	0.00	201	0.00
37.825	206	0.00	238	0.00
37.863	331	0.00	263	0.00

37.983	4551	0.04	697	0.01
38.213	12868	0.10	2789	0.03
38.330	1440	0.01	265	0.00
38.459	386	0.00	328	0.00
38.542	245	0.00	199	0.00
38.582	143	0.00	120	0.00
38.628	358	0.00	103	0.00
38.695	172	0.00	201	0.00
38.768	640	0.01	236	0.00
38.852	345	0.00	180	0.00
38.905	226	0.00	144	0.00
39.016	2801	0.02	490	0.01
39.145	243	0.00	202	0.00
39.193	234	0.00	218	0.00
39.258	576	0.00	259	0.00
39.403	737	0.01	201	0.00
39.428	193	0.00	162	0.00
39.468	193	0.00	71	0.00
39.504	890	0.01	244	0.00
39.677	211	0.00	98	0.00
39.833	11851	0.10	1846	0.02
39.978	505	0.00	186	0.00
40.034	199	0.00	160	0.00
40.070	236	0.00	169	0.00
40.120	159	0.00	151	0.00
40.147	169	0.00	208	0.00
40.209	333	0.00	151	0.00
40.252	1	0.00	26	0.00
40.261	213	0.00	197	0.00
40.302	411	0.00	273	0.00
40.336	294	0.00	186	0.00
40.381	517	0.00	177	0.00
40.458	253	0.00	205	0.00
40.478	33	0.00	142	0.00
40.552	130	0.00	180	0.00
40.564	93	0.00	175	0.00
40.597	129	0.00	175	0.00
40.644	264	0.00	219	0.00
40.701	123	0.00	194	0.00
40.736	243	0.00	213	0.00
40.778	187	0.00	212	0.00
40.815	203	0.00	152	0.00
40.855	70	0.00	89	0.00
40.882	138	0.00	160	0.00

40.912	322	0.00	227	0.00
40.964	232	0.00	202	0.00
41.012	224	0.00	201	0.00
41.053	214	0.00	196	0.00
41.079	118	0.00	97	0.00
41.106	262	0.00	170	0.00
41.162	254	0.00	189	0.00
41.213	346	0.00	202	0.00
41.248	216	0.00	154	0.00
41.283	68	0.00	139	0.00
41.314	214	0.00	189	0.00
41.345	260	0.00	185	0.00
41.436	220	0.00	196	0.00
41.468	154	0.00	198	0.00
41.493	330	0.00	179	0.00
41.567	457	0.00	204	0.00
41.598	277	0.00	223	0.00
41.657	171	0.00	168	0.00
41.684	197	0.00	189	0.00
41.745	568	0.00	295	0.00
41.971	11037	0.09	1216	0.01
42.150	245	0.00	177	0.00
42.173	150	0.00	179	0.00
42.202	161	0.00	177	0.00
42.254	161	0.00	189	0.00
42.285	209	0.00	148	0.00
42.347	262	0.00	164	0.00
42.362	139	0.00	141	0.00
42.398	312	0.00	182	0.00
42.454	342	0.00	225	0.00
42.478	275	0.00	133	0.00
42.547	296	0.00	276	0.00
42.628	298	0.00	201	0.00
42.690	371	0.00	231	0.00
42.755	133	0.00	187	0.00
42.773	183	0.00	205	0.00
42.852	537	0.00	510	0.01
42.916	166	0.00	145	0.00
42.956	418	0.00	143	0.00
43.037	276	0.00	164	0.00
43.081	303	0.00	231	0.00
43.127	263	0.00	256	0.00
43.157	756	0.01	274	0.00
43.228	366	0.00	221	0.00

43.267	508	0.00	276	0.00
43.312	412	0.00	245	0.00
43.343	372	0.00	230	0.00
43.382	340	0.00	230	0.00
43.423	420	0.00	264	0.00
43.475	308	0.00	248	0.00
43.516	271	0.00	159	0.00
43.553	218	0.00	140	0.00
43.633	113	0.00	209	0.00
43.653	157	0.00	171	0.00
43.703	404	0.00	289	0.00
43.728	269	0.00	193	0.00
43.766	238	0.00	238	0.00
43.809	461	0.00	233	0.00
43.849	433	0.00	261	0.00
43.887	230	0.00	236	0.00
43.911	375	0.00	229	0.00
43.978	451	0.00	213	0.00
44.031	322	0.00	227	0.00
44.075	277	0.00	204	0.00
44.121	288	0.00	251	0.00
44.167	488	0.00	297	0.00
44.215	245	0.00	363	0.00
44.229	170	0.00	214	0.00
44.254	269	0.00	183	0.00
44.309	419	0.00	273	0.00
44.324	286	0.00	245	0.00
44.387	1032	0.01	317	0.00
44.463	538	0.00	291	0.00
44.489	276	0.00	275	0.00
44.520	187	0.00	205	0.00
44.550	367	0.00	232	0.00
44.602	224	0.00	182	0.00
44.628	261	0.00	226	0.00
44.803	2356	0.02	425	0.00
44.829	547	0.00	295	0.00
44.910	611	0.00	198	0.00
44.997	283	0.00	208	0.00
45.040	247	0.00	217	0.00
45.067	346	0.00	245	0.00
45.103	141	0.00	223	0.00
45.132	250	0.00	268	0.00
45.160	298	0.00	192	0.00
45.222	395	0.00	190	0.00

45.330	450	0.00	605	0.01
45.357	242	0.00	208	0.00
45.386	172	0.00	198	0.00
45.423	259	0.00	229	0.00
45.439	557	0.00	258	0.00
45.524	266	0.00	253	0.00
45.583	388	0.00	202	0.00
45.626	155	0.00	163	0.00
45.647	143	0.00	141	0.00
45.681	179	0.00	144	0.00
45.715	168	0.00	208	0.00
45.786	106	0.00	166	0.00
45.907	506	0.00	212	0.00
45.956	531	0.00	239	0.00
46.004	189	0.00	177	0.00
46.042	275	0.00	182	0.00
46.079	361	0.00	215	0.00
46.151	358	0.00	243	0.00
46.216	519	0.00	284	0.00
46.243	421	0.00	215	0.00
46.307	339	0.00	215	0.00
46.333	392	0.00	231	0.00
46.362	418	0.00	233	0.00
46.408	276	0.00	283	0.00
46.438	464	0.00	233	0.00
46.503	365	0.00	225	0.00
46.528	184	0.00	257	0.00
46.563	378	0.00	233	0.00
46.618	455	0.00	223	0.00
46.667	249	0.00	222	0.00
46.725	577	0.00	302	0.00
46.774	458	0.00	275	0.00
46.807	550	0.00	329	0.00
46.843	686	0.01	304	0.00
46.898	572	0.00	366	0.00
46.930	384	0.00	320	0.00
46.965	576	0.00	287	0.00
47.001	634	0.01	333	0.00
47.057	494	0.00	400	0.00
47.090	488	0.00	316	0.00
47.135	623	0.01	326	0.00
47.157	485	0.00	272	0.00
47.208	392	0.00	347	0.00
47.280	1035	0.01	353	0.00

47.320	1456	0.01	345	0.00
47.441	918	0.01	340	0.00
47.499	311	0.00	288	0.00
47.539	580	0.00	315	0.00
47.607	996	0.01	304	0.00
47.662	434	0.00	286	0.00
47.695	562	0.00	260	0.00
47.752	457	0.00	232	0.00
47.831	626	0.01	254	0.00
47.875	420	0.00	238	0.00
47.938	878	0.01	261	0.00
47.991	217	0.00	272	0.00
48.040	801	0.01	261	0.00
48.131	647	0.01	285	0.00
48.171	373	0.00	250	0.00
48.294	892	0.01	264	0.00
48.324	453	0.00	307	0.00
48.400	1075	0.01	295	0.00
48.467	1211	0.01	348	0.00
48.537	1230	0.01	455	0.01
48.578	1205	0.01	549	0.01
48.615	1136	0.01	491	0.01
48.656	2313	0.02	423	0.00
48.790	201	0.00	298	0.00
48.813	377	0.00	351	0.00
48.843	258	0.00	222	0.00
48.888	521	0.00	270	0.00
48.933	228	0.00	218	0.00
48.966	278	0.00	180	0.00
